# Diabetes, Diabetic Complications, and Fracture Risk

**DOI:** 10.1007/s11914-015-0260-5

**Published:** 2015-02-04

**Authors:** Ling Oei, Fernando Rivadeneira, M. Carola Zillikens, Edwin H. G. Oei

**Affiliations:** 1 Department of Internal Medicine, Erasmus MC, University Medical Center, Rotterdam, The Netherlands; 2 Department of Epidemiology, Erasmus MC, University Medical Center, Rotterdam, The Netherlands; 3 Netherlands Genomics Initiative (NGI)-sponsored Netherlands Consortium for Healthy Aging (NCHA), Rotterdam, The Netherlands; 4 Department of Internal Medicine, IJsselland Hospital, Capelle aan den IJssel, The Netherlands; 5 Department of Radiology, Erasmus MC, University Medical Center, Rotterdam, The Netherlands

**Keywords:** Diabetes mellitus, Osteoporosis, Fracture risk, Diabetic complications, Diabetes-related bone disease, Bone metabolism

## Abstract

Diabetes and osteoporosis are both common diseases with increasing prevalences in the aging population. There is increasing evidence corroborating an association between diabetes mellitus and bone. This review will discuss the disease complications of diabetes on the skeleton, highlighting findings from epidemiological, molecular, and imaging studies in animal models and humans. Compared to control subjects, decreased bone mineral density (BMD) has been observed in type 1 diabetes mellitus, while on average, higher BMD has been found in type 2 diabetes; nonetheless, patients with both types of diabetes are seemingly at increased risk of fractures. Conventional diagnostics such as DXA measurements and the current fracture risk assessment tool (FRAX) risk prediction algorithm for estimating risk of osteoporotic fractures are not sufficient in the case of diabetes. A deterioration in bone microarchitecture and an inefficient distribution of bone mass with insufficiency of repair and adaptation mechanisms appear to be factors of relevance. A highly complex and heterogeneous molecular pathophysiology underlies diabetes-related bone disease, involving hormonal, immune, and perhaps genetic pathways. The detrimental effects of chronically elevated glucose levels on bone should be added to the more well-known complications of diabetes.

## Introduction

Diabetes and osteoporosis are common diseases with increasing prevalences in the aging population. There is growing evidence corroborating that diabetes mellitus influences the skeletal metabolism. Decreased bone mineral density (BMD) and increased fracture risk have fairly consistently been observed in type 1 diabetes mellitus patients [1••]. This review will primarily focus on type 2 diabetes. Contradictory results with higher, lower, or similar values for BMD observed in persons with type 2 diabetes compared to control subjects have been reported across individual and relatively small studies with diverse designs [2–5]. Nevertheless, several lines of evidence arising from meta-analytical efforts suggest that individuals with type 2 diabetes have generally higher BMD levels at the femoral neck, hip, and spine than persons without diabetes, independently of gender or body mass index (which is usually higher in subjects with type 2 diabetes and discussed in further detail below) [1••, 6•]. The between-study heterogeneity was very high and originated at least in part from differences in design and possibly diabetes definition across studies. Nonetheless, the meta-regression of the results across studies showed that younger age, male gender, higher body mass index, and higher hemoglobin A1c (HbA_1C_) were positively associated with higher BMD levels in individuals with type 2 diabetes.

## Higher Fracture Risk Despite a Higher Bone Mineral Density in Type 2 Diabetes

Based on evidence in non-diabetics, the higher levels of BMD should be protective against fracture; this association seems somewhat different in type 2 diabetes [2, 7, 8••, 9]. Using data from the prospective Rotterdam Study cohort, De Liefde et al. were among the first to show that individuals with type 2 diabetes have 69 % higher risk of non-vertebral fractures than those without diabetes despite having higher BMD at the femoral neck and lumbar spine [9]. The aforementioned meta-analysis by Vestergaard et al. found summary estimates for hip fracture risk of 6.9 in type 1 and 1.4 in type 2 diabetes compared to subjects without diabetes, respectively [1••]. Schwartz and colleagues established in a meta-analysis based on three prospective observational studies with adjudicated fracture outcomes (Study of Osteoporotic Fractures; Osteoporotic Fractures in Men Study; and Health, Aging, and Body Composition Study) that in type 2 diabetes patients, the fracture risk was higher for a given BMD and age as compared with participants without diabetes and, most importantly, that the World Health Organization’s fracture risk assessment tool (FRAX) underestimates osteoporotic fracture risk in individuals with diabetes [8••]; similar work by Giangregorio et al. in the Canadian Manitoba Bone Density Program illustrated how diabetes as a risk factor is necessary to be considered for inclusion in future iterations of FRAX [10]. Even though most of the work has been done in populations of European background, similar relationships have been observed across different ethnicities, particularly in relation to increased risk of vertebral fractures [11–13].

Many studies have shown a difference in population characteristics between type 2 diabetic patients and healthy controls [3, 9, 14, 15]. In these studies, diabetic study participants tend to be older, have a higher body mass index (BMI) or weight, increased insulin levels, less physical exercise, higher alcohol consumption, and they usually smoke more and more often. Also, the use of diuretics is more common in diabetes, and particularly loop diuretics (e.g., furosemide) may be associated with decreased BMD and increased risk of fractures through increasing urinary calcium excretion and osteoclastic bone resorption [16], while thiazides are associated with higher BMD and lower fracture risk [17, 18]. Further, the use of anti-diabetic thiazolidinediones has been reported to increase fracture risk [19]. Patients with diabetes fall more often, which can be a consequence from suffering from suboptimal physical fitness, neuropathy, retinopathy, or sarcopenia [20]. Alternatively, insulin users with low HbA_1C_ levels are reported to fall more, likely as a consequence of hypoglycemia [21]. These characteristics might influence bone metabolism and fracture risk; nevertheless, statistical analyses with corrections in aforementioned studies suggest independence of the differences in BMD and fracture risk from these measured confounders [3, 9, 14, 15] such as risk of falling [9, 14].

## Relation of Diabetes Regulation with Fracture Risk

Some studies evaluating the relationship between glycemic control based on fasting blood glucose and fracture risk have found conflicting results [26–30]. Other factors that do seem to matter are the use of insulin and disease duration. Among these studies is an investigation by Ivers et al. [22] which found that fasting blood glucose greater than 7 mmol/L, disease duration longer than 10 years, insulin treatment, and the presence of diabetic retinopathy were associated with increased risk of all types of fractures. The oral glucose tolerance test (OGTT) remains the gold standard for distinguishing diabetes mellitus (pre-glucose load or post-glucose load challenge serum glucose level of 11.1 mmol/l or higher) and impaired glucose tolerance (pre-glucose load or post-glucose load challenge serum glucose level from 7.8 to 11.1 mmol/l) [23]. In the Rotterdam Study, subjects with type 2 diabetes and impaired glucose tolerance were both found to have higher BMD, whereas contrary to those with impaired glucose tolerance, patients with type 2 diabetes had higher fracture risk, particularly those on anti-diabetic medication [9]. Nevertheless, HbA_1C_ is a better indicator than serum glucose for long-term diabetes control and is therefore considered the main parameter in clinical practice.

Higher HbA_1C_ reflects a higher average plasma glucose concentration over a prolonged period, in the order of weeks. We observed in Rotterdam Study data that poor glycemic control based on an HbA_1C_ cut-off of 7.5 % (58 mmol/l) in type 2 diabetes is associated with higher all types of fracture risk, higher BMD, and thicker femoral cortices in narrower bones [24••]. Intriguingly, different HbA_1C_ thresholds were applied in various studies, possibly due to heterogeneity in effects and study population. Similar to our observations, the Atherosclerosis Risk in Communities (ARIC) Study found that type 2 diabetes was significantly and independently associated with increased risk of fracture. In this study, an increased risk of fracture of 1.87 times was observed among persons treated with insulin and an increased risk of 1.63 times among persons with diagnosed diabetes with HbA_1C_ ≥8 % (64 mmol/l) as compared to those individuals with HbA_1C_ below 8 % [25••]. Kanazawa et al. found that obese Japanese men with type 2 diabetes and HbA_1C_ of 9 % and above had three times increased risk of vertebral fracture than men with diabetes but normal BMI, despite equal or higher BMD [26]. Strotmeyer et al. found that older white and black adults with type 2 diabetes in the Health ABC Study had 1.6 increased risk of fracture [13]. However, when comparing diabetes patients with and without fractures, poor glycemic control (threshold of HbA_1C_ 7 % (53 mmol/l)), longer disease duration, and insulin use were not significantly different. Forsén et al. [26] found that fracture risk was higher in Norwegian subjects with a disease duration longer than 5 years and insulin use, but failed to demonstrate any effect on fractures using a high cut-off of HbA_1C_ 9.5 % (80 mmol/l). Yet, this cut-off was very high and a consequent lack of study power cannot be ruled out.

## Pathophysiology

A highly complex and heterogeneous molecular pathophysiology seems to underlie fracture risk in diabetes-related bone disease. One of the factors that have been found detrimental is advanced glycation end products (AGEs). AGEs are generated by the sequential non-enzymatic addition of carbohydrate molecules to protein amino groups [27]. AGEs accumulate in various tissues including bone [28, 29], kidney, and coronary arteries [30]. This may result in the development of diabetic complications through increased inflammation, interference with normal tissue function, and cellular damage. Pentosidine is one of the well-known AGEs, and the accumulation of pentosidine in cortical and trabecular bone is negatively associated with bone strength [28, 29, 31]. Histopathological analyses comparing bone samples from femoral neck fracture cases with post-mortem controls revealed a higher extent of hydroxylation and higher pentosidine content [32, 33]. Furthermore, Yamamoto et al. showed that individuals with type 2 diabetes suffering from vertebral fractures have increased serum levels of pentosidine [34•], while higher levels of the endogenous secretory receptor for AGEs (esRAGE), acting as a decoy receptor binding AGEs, have protective effects on fracture risk in diabetes [35]. esRAGE is the most prevalent splice variant of RAGE, while the most common form is full-length RAGE [36], which possesses a transmembrane domain and is therefore able to transduce signals as a membrane-bound receptor [37]. Seemingly, full-length RAGE has a role in bone remodeling by regulating osteoclast function possibly through integrin signaling and bone mass given that mice lacking RAGE have increased bone mass and BMD and decreased bone resorptive activity in vivo [38].

Insulin levels could mediate in part a positive association between type 2 diabetes and elevated BMD. Individuals with type 2 diabetes usually have an excess of insulin, and those with worse glucose control have the highest serum levels [24••]. Physiologically, insulin has an anabolic effect on bone due to its structural homology to insulin-like growth factor-I (IGF-I) by interacting with the IGF-I receptor present on osteoblasts [39]. The IGF-I signaling pathway is crucial for bone acquisition and bone remodeling [40]. The lower concentrations of serum IGF-I levels are associated with the presence of and a higher number of prevalent vertebral fractures in postmenopausal women with type 2 diabetes [41, 42]. Additionally, novel data from a mouse study with osteoprogenitor-selective ablation of the insulin receptor suggest that insulin receptor malfunction itself may directly lead to biomechanical microarchitecture alterations in both cortical and trabecular bone [43•]. Furthermore, there is evidence that insulin receptor signaling promotes the differentiation of osteoblasts and enhances the production and activation of osteocalcin [44, 45].

Osteocalcin is an osteoblast-specific secreted protein that regulates hydroxyapatite size and shape through its vitamin K-dependent, gamma-carboxylated form, thereby reflecting bone remodeling and, in particular, bone formation [46]. The metabolic roles of osteocalcin have been identified in animal studies, including increasing insulin secretion and sensitivity [47]. The regulation of insulin sensitivity by osteocalcin may be either direct or indirect, via the adipocyte-derived hormone adiponectin (discussed below) [46]. Osteocalcin has also been found to be negatively correlated with HbA_1C_ as a marker of glycemic control in type 1 and type 2 diabetes [26]. Osteocalcin knock-out mice display glucose intolerance and insulin resistance with a concomitant slight increase in bone density [48••]. In bone and serum, osteocalcin is incompletely carboxylated (undercarboxylated osteocalcin), and it is this uncarboxylated form that has been negatively implicated in energy metabolism and glucose control in both mice and humans [45, 47]. Higher undercarboxylated osteocalcin may be linked to increased risk of hip fracture [49•], where calcium and vitamin D2 suppletion was able to normalize the undercarboxylated osteocalcin levels [50••]. The underlying mechanism is largely unknown; it is known that 1,25-dihydroxyvitamin D enhances the transcription of osteocalcin by means of the gene possessing a vitamin D-responsive element [51], but whether vitamin D might directly influence the γ-carboxylation reaction of osteocalcin remains unclear [52, 53]. Cardiovascular disease including atherosclerosis is more common in type 2 diabetes mellitus; studies carried out so far suggest that abdominal aortic calcification is more common in diabetics [54]. In Asian women, it has been observed that osteocalcin significantly correlated with aortic calcification, which again is associated with a threefold increased risk of vertebral fractures [55].

Adipokines are cell signaling proteins secreted by adipose tissue and include for instance leptin, adiponectin, and resistin. The release of these adipokines leads to a chronic subinflammatory state that could play a central role in the development of insulin resistance and type 2 diabetes [56]. It has been observed that plasma leptin concentrations are higher in obese persons with diabetes than in healthy controls [57]. Leptin induces bone growth by stimulating osteoblast proliferation and differentiation [58–60], and it has also been shown to inhibit osteoclastogenesis through reducing RANK/RANK-ligand production and increasing osteoprotegerin [61, 62]. Plasma leptin concentrations have been found inversely related with BMD in cross-sectional studies [63–65]. Further, higher leptin levels were associated with a lower prevalence of fracture in some cohorts [66], though the effect may not be as clear in individuals aged 70 to 79 years from the Health Aging and Body Composition Study [67]. Some reports indicate that circulating adiponectin and resistin levels are reduced in diabetes [68]. Adiponectin is expressed in osteoblasts and osteoclasts [69], and adiponectin seems to influence differentiation from mesenchymal progenitor cells into osteocytes or adipocytes, yet the effects on bone metabolism remain unclear [70, 71]. After the adjustments of measures of body fat, each doubling of adiponectin is associated with a 2–3 % decrease in BMD [72], and higher adiponectin levels may be a risk factor for increased fracture risk [67]. The gut-derived peptide hormone ghrelin has been shown to modulate osteoblast differentiation and function, both directly and perhaps also through the regulation of the growth hormone–insulin-like growth factor axis and through interaction with leptin ghrelin has a role in modulating bone structure [73]. A systematic review and meta-analysis by Biver et al. concluded that the most relevant adipokines influencing BMD and fracture risk are indeed leptin and adiponectin, whereas no convincing data are available for resistin, visfatin, or gut-derived ghrelin [74].

The role of inflammation in the pathogenesis of type 2 diabetes, as touched upon before, and associated complications is now well established [75]. C-reactive protein (CRP) is an extremely sensitive marker of systemic inflammation produced mainly by the liver under the stimulation of macrophage- and adipocyte-derived proinflammatory cytokines, principally interleukin-6 (IL-6) [76]. Elevated levels of CRP are described in persons with type 2 diabetes; however, it is not clear if they are related to the presence of obesity, diabetes, or both [77]. Studies in general populations have found lower BMD [78, 79], lower hip geometrical bending strength [80•], and an increased risk of fracture [80•, 81] for higher CRP levels, which intriguingly appeared to be independent of BMD or trabecular microarchitecture [82]. Some studies explicitly indicate a relationship between CRP and complications of diabetes [83–86]; nonetheless, evidence is lacking for a direct mechanism, and CRP may very well merely be a marker of the ongoing inflammation [80•, 87–89].

## Shared Genetic Factors Between Diabetes and Bone Disease

A genome-wide association study (GWAS) meta-analysis for gene expression levels in relation to type 2 diabetes as the phenotype of interest including 1175 case–control microarrays showed a significantly differential gene expression of osteopontin (OPN), also known as phosphoprotein 1 (SPP1) or bone sialoprotein I (BSP-I) [90]. This same investigation brought forward that osteopontin is a ligand for the most prominent top hit of this genome-wide screening being the immune cell receptor CD44 and that the expression profiles of CD44 and osteopontin are frequently coordinately dysregulated, especially in adipose tissue. The gene-encoding osteopontin maps to the 4q22.1 locus, which has frequently appeared as a femoral neck-BMD and lumbar spine-BMD locus in large-scale meta-analyses and contains many bone-active genes [91•, 92–94]. Osteopontin is an extracellular structural protein in bone able to bind strongly to calcium crystals [95]. It has been proposed that osteopontin is an important factor in bone remodeling [96], which may be by anchoring osteoclasts to the mineral matrix of bones [97]. In addition, osteopontin enhances B lymphocyte proliferation and immunoglobulin production and is chemotactic for many immune cell types including macrophages, dendritic cells, and T cells [98]. Osteopontin null mice of all ages display a bone phenotype probably mediated by altered osteoclast activity, protecting them from developing osteoporosis [99]. Fascinatingly, wild-type mice exposed to a high-fat diet exhibit increased plasma osteopontin levels with elevated expression in macrophages recruited into adipose tissue, while on the other hand, obese osteopontin null mice exhibit decreased markers of inflammation with less macrophage infiltration into adipose tissue, display improved insulin sensitivity, and are seemingly protected from the effects of diet-induced obesity on body composition or energy expenditure [100]. Altogether, this suggests a key role for osteopontin in the development of age-related osteoporosis and the link of obesity to the development of insulin resistance and possibly type 2 diabetes.

A GWAS meta-analysis targeting copy number variations (CNV), which are a type of structural variants of the genome in which large (>1 kb) segments of the genome are either lost or duplicated, found evidence that a deletion in the 6p25.1 locus predisposes to risk of all types of fracture [101•]. The deletion is located in an intergenic region in the subtelomeric region of chromosome 6p in the proximity of the peroxisomal D3, D2-EnoylCoA Isomerase (*PECI*) gene which codes for an enzyme relevant for the metabolism of fatty acids. *PECI* was first cloned by using pooled antisera from autoimmune diabetes patients [102]. The increased risk seen with individuals with the 6p25del may be mediated by co-morbidity with diabetes, yet more studies are needed to convincingly replicate the potential association of this copy number variant with fracture risk and elucidate the underlying functional mechanism.

The association between BMD, type 2 diabetes, and glycemic traits [103] was also tested in the context of pleiotropic relations by members of the Genetic Factors of Osteoporosis (GEFOS) and Meta-Analyses of Glucose and Insulin-related traits (MAGIC) consortia. None of the BMD single nucleotide polymorphisms (SNPs) reached the a priori *P* value threshold corrected for multiple testing, except a SNP at the *ITGA1* locus. This marker was found associated with type 2 diabetes, serum insulin levels, β-cell function, and glucose tolerance. Null *ITGA1* mice have impaired fracture healing and cartilage remodeling [104], although it is not yet clear what role this gene product has on BMD or bone structure.

## Bone Geometry

Our data on hip bone geometry in the Rotterdam Study showed that individuals with inadequately controlled diabetes have persistently thicker cortices in narrower femoral necks than those with adequately controlled diabetes or those without diabetes [24••]. A lesser tendency to undergo physiological bone expansion (periosteal apposition), i.e., a process in which a limited amount of bone mass is efficiently redistributed, could be inferred from narrower bone diameters in these individuals. This led us to propose that changes in microarchitecture (i.e., microcracks and cortical porosity) could be underlying the increased risk of fractures observed in inadequately controlled diabetics. A peripheral quantitative computed tomography (pQCT) investigation in the Osteoporotic Fractures in Men Study found that participants with type 2 diabetes displayed greater volumetric bone mineral density (vBMD) but a smaller bone area at both the distal tibia and radius, which resulted in a bone strength which was particularly low relative to body weight [105]. As described by Ahlborg et al. [106], a process of rapid physiological bone expansion occurs in women after menopause, highlighting a complex interplay of hormones such as estradiol, IGF-I, and insulin [107, 108]. Considering the known anabolic effects of IGF-I and insulin on bone and periosteal expansion, it can be expected that the altered insulin–IGF-I–growth hormone axis (lower bioavailability of IGF-I) may also contribute to the observed geometrical alterations observed in inadequately controlled diabetes, as a lack of periosteal apposition and bone repair. Since such differences in geometry are accentuated at older ages, we previously postulated that an accumulation of microcracks with time may well be a skeletal complication of inadequately controlled diabetes resulting in impaired bone repair, decreased bone remodeling, high BMD, and increased risk of fracture [24••]. There is a growing body of evidence for the deterioration of bone microarchitecture in type 2 diabetes leading to a porous skeleton susceptible to fracture. Burghardt et al. applied a novel derivative of cortical porosity for high-resolution peripheral quantitative computed tomography (HR-pQCT) and reported that the cortical porosity in type 2 diabetic patients is up to twice that of controls at the radius [109••]. Subsequently, Patsch et al. compared type 2 diabetes patients with fragility fractures to patients with diabetes without fractures and controls with and without fractures [110••]. The investigators showed nicely that the cortical porosity is specific to those type 2 diabetes patients that have a fracture. Similarly, the trabecular bone score (TBS) is a measure of bone texture that can be derived from DXA, which correlates with 3D parameters of bone microarchitecture [111]. One of the first studies utilizing this invention demonstrated that TBS is lower at the lumbar spine in diabetes-related bone disease [112]. The results of these investigations provide a potential explanation for the inability of standard DXA measures to explain the elevated fracture incidence in patients with diabetes presenting with higher BMD and apparently stronger bone geometry.

Recently, researchers have started to examine bone marrow fat composition, regarding presence and types of hydrogen bonds, where unsaturated fats contain at least one double bond, and saturated fats have the maximum number of hydrogens bonded to carbons. The radiological research group of Dr. Link has demonstrated in their combined quantitative computed tomography (QCT) and magnetic resonance (MR) spectroscopy studies that the prevalence of fragility fractures is associated with lower unsaturation levels and higher saturation levels of bone marrow fat, in which the participants with diabetes with fractures have the lowest marrow unsaturation and highest saturation [113]. In contrast to controls without diabetes, higher mean vertebral bone marrow fat content is significantly correlated with visceral adipose tissue and HbA_1C_ in persons with type 2 diabetes, representing worse metabolic profiles [114]. The concept of high-saturated fat-associated adipose inflammation and insulin resistance has been proposed; however, underlying molecular mechanisms remain to be elucidated.

Reference point indentation [115, 116] allows minimally invasive measurements of bone material properties of human bone in vivo by microindentation, which is correlated with the risk of osteoporotic fractures [117, 118]. Recently, Farr et al. showed that patients with type 2 diabetes have reduced serum markers of bone turnover and lower bone material strength at the tibia than age-matched controls without diabetes [119••]. Further, in this same study, the average HbA_1C_ level over the previous 10 years was negatively correlated with bone material strength [119••], supporting the contention recognizing the skeleton as another important target tissue subject to diabetic complications [24••].

## Therapeutic Options

Not only are patients with diabetes at increased risk for fractures, but they also are prone to impaired bone healing after fracture [120]. In usual fracture healing, serum concentrations of biomarkers such as alkaline phosphatase, IGF-I, and osteocalcin peak in the first few weeks of recovery [121, 122] and decrease again thereafter, but possibly in disturbed consolidation, these levels remain elevated for an even longer time [123]. An experimental study using the diabetic Zucker (fa/fa) rat model with creation of femoral defects demonstrated that the administration of parathyroid hormone (PTH) could partially reverse the adverse skeletal effects of diabetes on bone defect [124].

Systematic screening for complications and fall prevention efforts, along with calcium and vitamin D repletion and adequate physical activity, represents the mainstay of fracture prevention in patients with diabetes. Nonetheless, we should mention that the controversy regarding the anti-fracture efficacy versus the side-effect profile of calcium supplements in general is still unresolved [125–127]. A few meta-analyses with different methodologies have been published on this topic to date yielding conflicting results [128–131], of which the investigation by Bolland et al. suggested an increased risk of myocardial infarction (MI) and possibly stroke in men and women together for calcium supplements, particularly without co-administered vitamin D [128]. These specific potential side effects of calcium supplements may be of particular importance in patients with diabetes as they are already at increased risk of cardiovascular disease complications; however, no studies have been performed in this area yet. As discussed above, the current FRAX risk score underestimates fracture risk in patients with diabetes, which leads to undertreatment of the diabetic individuals that are actually at increased fracture risk. Anti-catabolic drugs (raloxifene, bisphosphonates, denosumab) might be effective, but on the basis of pathophysiological evidence that suggests low bone formation in the aforementioned research in model organisms [124], osteo-anabolic therapies such as teriparatide might represent an important therapeutic option for diabetes-related bone disease [132]. More studies including randomized controlled trials in this area are needed.

## Strength of Evidence

The evidence outlined in this review includes studies in humans and animals. Animal studies cited are mostly knock-out mice experiments. Human studies include observational studies of varying sizes, meta-analyses summarizing these results, and a few randomized controlled trials of generally smaller sample sizes. At present, it may not be very well possible to grade the evidence; replication studies in this field are desirable.

## Conclusion

In conclusion, the detrimental effects of diabetes on bone should be added to the more well-known complications of diabetes. A deterioration in bone microarchitecture and an inefficient distribution of bone mass with insufficiency of repair and adaptation mechanisms in combination with increased risk of falling all lead to an elevated fracture risk as skeletal complications of diabetes. Improved risk prediction with epidemiological determinants and integration of novel biochemical and imaging biomarkers will be necessary to correctly and timely diagnose those individuals at increased risk. More research is needed to unravel the pathophysiology underlying diabetes-related bone disease, which may eventually contribute to preventative and curative therapies.
